# Nd:YAG membranotomy for sub-inner limiting membrane hemorrhage: a case report

**DOI:** 10.22336/rjo.2021.15

**Published:** 2021

**Authors:** Jaime Larrea, Javier Lacorzana, Nicolás Rivera-Valdivia, Diego Bueso, Carlos Abdala-Caballero

**Affiliations:** * Retina and Vitreous Department, Grupo Oftalmológico Abdala-Figuerola AF, Barranquilla, Colombia; ** Department of Ophthalmology, Virgen de las Nieves University Hospital, Granada, Spain; *** Doctoral Program in Clinical Medicine and Public Health, University of Granada, Granada, Spain; **** Retina and Vitreous Department, Grupo Oftalmológico Abdala-Figuerola AF, Barranquilla, Colombia

**Keywords:** sub-inner limiting membrane hemorrhage, Valsalva maneuver, Nd:YAG laser membranotomy

## Abstract

**Case Description:** A 16-year-old male patient presented with a 12-days sudden painless loss of vision in his left eye after diving in a lake. Best corrected visual acuity (BCVA) in the left eye was counting fingers. Anterior segment was unremarkable. Fundoscopy in the left eye revealed a pre-retinal hemorrhage in the macular region and swept-source ocular coherence tomography (SS-OCT) confirmed the location in the sub-inner limiting membrane (ILM) space. An Neodymium:YAG (Nd:YAG) laser membranotomy was performed the next day in order to drain the hemorrhage into the vitreous cavity. A couple of days after, the BCVA in the left eye improved to 20/ 25, at fundoscopy the blood being almost reabsorbed and the SS-OCT showing a resolution of the sub-ILM hemorrhage.

**Discussion:** Due to Valsalva retinopathy, sub-ILM hemorrhage may lead to a sudden painless vision loss. Spontaneous resolution of the hemorrhage is possible but absorption may take a long time. During this period, intraretinal tissue migration and proliferation may lead to permanent structural damage. Posterior vitrectomy is a treatment option but the fact that it is an invasive procedure fuels the search for less invasive treatment methods and Nd:YAG laser membranotomy fits this place.

**Conclusion:** Given the excellent results and low complication rates, Nd:YAG laser membranotomy is highly recommended to treat this condition as it offers a simple, relatively safe and a non-invasive treatment option for drainage of sub-ILM hemorrhages.

## Introduction

Preretinal hemorrhages commonly occur at the interface between the posterior hyaloid and the ILM. In a few cases, they appear in the superficial retina between the ILM and the retinal nerve fiber layer. These demarcated hemorrhages show a predilection for the macular region and consequently lead to a sudden visual loss [**1**]. Sub-ILM nature of the hemorrhage remains difficult to distinguish clinically, the most common causes are Valsalva retinopathy (VR) [**1**], as in our case, and Terson’s syndrome [**2**], each of them with different pathophysiology and less frequently proliferative diabetic retinopathy, macroaneurysm, vein occlusion, trauma, and hematologic disorders [**3**]. 

Management options include observation, Neodymium:YAG (Nd:YAG) laser membranotomy (LM) and pars plana vitrectomy (PPV) [**3**]. PPV is a treatment option but the fact that it is an invasive procedure fuels the search for less invasive treatment methods and LM fits this place, as it offers a safe, simple, low complications rate, inexpensive and non-invasive treatment option for drainage of sub-ILM hemorrhages. We reported a case of the successful treatment with LM of a sub-ILM hemorrhage due to VR confirmed by SS-OCT.

## Case Description

**Fig. 1:**
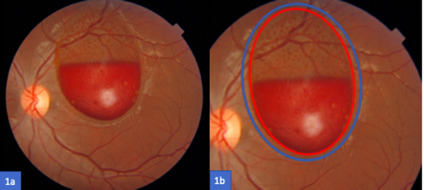
**a.** Fundus color photography demonstrating the pre-retinal hemorrhage; **b.** A close-up of Fig. 1a; The blue ring stands for the hyaloid ad the red ring for the ILM

A 16-year-old male patient presented with a 12-days sudden onset of painless loss of vision in his left eye (OS) after diving in a lake. BCVA in OS was counting fingers. Anterior segment was unremarkable, fundoscopy showed a well-circumscribed pre-retinal hemorrhage with a fluid level in the posterior segment (**Fig. 1a,b**).

**Fig. 2:**
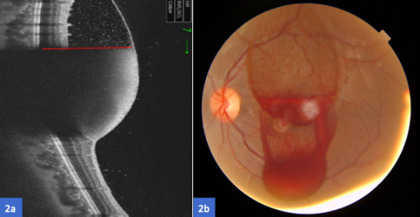
**a.** SS-OCT before the membranotomy demonstrates the fluid level with the red line; **b.** Fundus color photography minutes after the Nd:YAG laser membranotomy, showing how the blood moves to the inferior region of the vitreous cavity

**Fig. 3:**
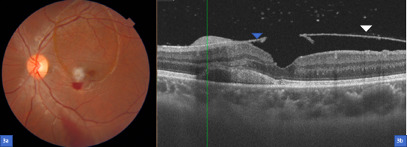
**a.** Fundus color photography a couple of days after the Nd:YAG laser membranotomy with the blood almost reabsorbed, just a few remnants of vitreous hemorrhage in the inferior region; **b.** SS-OCT showing the resolution of the sub-ILM hemorrhage and just above the fovea the place where the laser shot was done. Also, an intraretinal hemorrhage can be seen in the external layers; the blue arrowhead shows the ILM and the white arrowhead the hyaloid

SS-OCT confirmed the location in the sub-ILM space (**Fig. 2a**). An LM was performed the next day in order to drain the sub-ILM hemorrhage into the vitreous cavity. Two laser shots with a power of 2.7 mJ were done, aimed at the lower edge of the hemorrhage and blood moved to the inferior region of the vitreous cavity (**Fig. 2b**). A couple of days after, the BCVA in the OS improved to 20/ 25. At fundoscopy, the blood was almost reabsorbed (**Fig. 3a**) and the SS-OCT showed resolution of the sub-ILM hemorrhage (**Fig. 3b**).

## Discussion

Sub-ILM hemorrhage is a collection of blood between ILM and retinal nerve fiber layer [**4**], and, as mentioned before, VR is the most common cause. VR was first described by Thomas Duane in 1972. It has been reported to occur with straining and physical activities, most commonly during coughing, weight lifting [**4**], aerobic exercise, end-stage labor, constipation, among others [**5**]. The present case was a healthy young male like is typically seen [**4**], who developed VR after holding his breath while diving.

VR occurs as a result of a Valsalva maneuver [**5**,**6**], which consists in a forcible expiration against a closed glottis that produces a sudden rise in venous blood pressure due to the rise in intrathoracic or intra-abdominal pressure. Incompetent valves in the venous system of the head and neck allow transmission of thoracic and abdominal pressure into the eye [**5**]. This sudden rise of blood pressure leads to a sudden increase of intraocular venous pressure, which results in a rupture of superficial retinal capillaries, most commonly at the macular area [**6**] and this may be attributed to the absence of firm attachment of the posterior hyaloid and the ILM in the macular region [**7**] causing a hemorrhagic detachment of the ILM, which acts as a barrier preventing its spread to the subhyaloid space [**5**]. It usually presents unilateral but, in some cases, it is bilateral [**6**]. The presentation is usually with a sudden drop in vision after doing one activity previously mentioned in some cases floaters or even total loss of vision [**6**]. It presents in the macular region as a dome-like elevation. Generally, it is an isolated and self-limited event [**5**]. The blood is usually localized either in the sub-ILM space or the sub-hyaloid space. If the blood is trapped in both, it presents a “double-ring sign”. The outer blood ring represents the subhyaloid space and the inner ring the sub-ILM space [**6**].

Treatment options include observation, LM and PPV. If observation is chosen, we should advise the patient not do strenuous activities and to sleep in a sitting position to intend blood settling, which usually improves visual acuity [**5**]. Spontaneous resolution is possible but absorption may take a few weeks to months. During this period, intraretinal tissue may migrate and proliferate leading to a permanent structural damage because of the contact of the retina with haemoglobin and iron, causing a toxic damage to the retina and reducing visual function, which may be irreversible [**5**].

With time the sub-ILM hemorrhage may dehemoglobinize giving a yellowish and later white color to the hemorrhage. Once the hemorrhage clears, the only sign seen is a formation of a sub-ILM cavity. A clinical clue to previous sub-ILM bleed is the presence of brownish pigments (blood products) at the margin of the ILM detachment. This detached ILM usually reattaches at some point [**8**]. Other complications of longstanding intraocular blood persistence include cataract, epiretinal membranes [**9**], glaucoma, retinal detachment, and amblyopia if present in children [**1**].

If a quick recovery is desired observation may not be the best treatment option. This is particularly important as most of the patients belong to a younger age group and require a rapid visual rehabilitation in order to be able to continue working. Therefore, PPV with ILM peeling and LM could be the best option for these patients. Treatment with vitrectomy has shown to result in a significant and immediate visual improvement. The most common complications are increased intraocular pressure and cataract [**1**]. As a high number of patients are young, less invasive treatment methods that do not develop cataract are preferred, and LM fits in this place. Also, compared to surgical treatment in an operating room, it reduces the recovery time and lowers health care costs [**7**]. 

Drainage of a premacular subhyaloid haemorrhage and sub-ILM haemorrhage with Nd:YAG laser (1064 nm) was described by Faulborn in 1988, for an eye with diabetic retinopathy [**5**,**10**]. The recommended power of the LM varies between 2.2 to 10.5 mJ [**10**]. In our case we used a power of 2.5 mJ and two laser shots and it was enough to drain the sub-ILM haemorrhage. Using these powers helps to increase the cushion effect of the hemorrhage in order to avoid inadvertent retinal damage by the photodisruptive laser [**5**,**10**]. The site of membranotomy is recommended away from large blood vessels, away from the fovea, at the inferior margin of the hemorrhage [**7**,**10**]. The suggested laser treatment is only recommended if the size of the hemorrhage is beyond 3 DD, but other authors like Kim [**7**] reported that in small size hemorrhages the LM also goes well. Complications of laser therapy include iatrogenic retinal tear, hemorrhage into subretinal and choroidal space, macular hole, retinal detachment [**7**,**10**,**11**] and epiretinal membrane formation, this last one being described by Kwok et al. in 2003, when they reported the histologic findings of a case with VR operated for epiretinal membrane formation 10 months after Nd:YAG LM [**9**]. 

Short term results of the LM were studied by Khan et al. They reported a case series of 17 patients who underwent LM for Valsalva retinopathy. Most eyes demonstrated good visualization of the macula within one month. No post laser complications were noted at 6 months follow-up [**11**]. The long-term results of Nd:YAG LM were also studied. Durunkan et al. described a case series of 16 patients with 24 months of follow-up with premacular hemorrhages, who underwent LM. At the end of the study, all patients improved visual acuity to 20/ 20, and had no evidence of retinal or choroidal damage [**5**]. 

## Conclusion

The literature review and our study confirmed the good functional results for patients treated for VR by LM. Furthermore, very few side effects are reported with this type of treatment compared with the natural course of the disease, which may certainly regress spontaneously, but carries a risk of secondary preretinal fibrosis. We highly recommend the use of LM in cases of young patients, like ours, in which a rapid visual restoration was permitted, without any complications, because it was inexpensive to the patient and he avoided an invasive procedure at his young age, that would most likely have developed into a cataract within the years.

**Conflict of Interest**

The authors declare no conflict of interest.

**Informed Consent and Human and Animal Rights statements**

Informed consent has been obtained from all individuals included in this study.

**Authorization for the use of human subjects**

Ethical approval: The research related to human use complies with all the relevant national regulations, institutional policies, is in accordance with the tenets of the Helsinki Declaration, and has been approved by the Ethics Committee of Grupo Oftalmológico Abdala-Figuerola AF, Barranquilla, Colombia

**Acknowledgements**

None.

**Sources of Funding**

None.

**Disclosures**

None of the authors has any financial interest to disclose. 
